# Machine Diagnostics and Machine Phenotyping of Migraine: A HUNT Study

**DOI:** 10.1212/WNL.0000000000218076

**Published:** 2026-06-16

**Authors:** Antonios Danelakis, Håkon Kvisle Abildsnes, Fahim Faisal, Marte-Helene Bjørk, Dominic Giles, Knut Hagen, for the International Headache Genetics Consortium, Tjaṧa Kumelj, Manjit Matharu, Parashkev Nachev, Erling Tronvik, Bendik S. Winsvold, Anker Stubberud

**Affiliations:** 1 NorHead Norwegian Centre for Headache Research, NTNU Norwegian University of Science and Technology, Trondheim, Norway;; 2 Department of Computer Science, NTNU Norwegian University of Science and Technology, Trondheim, Norway;; 3 Department of Neuromedicine and Movement Science, NTNU Norwegian University of Science and Technology, Trondheim, Norway;; 4 Department of Clinical Medicine, University of Bergen, Bergen, Norway;; 5 Department of Neurology, Haukeland University Hospital, Bergen, Norway;; 6 UCL Queen Square Institute of Neurology, University College London, London, United Kingdom;; 7 Neuroclinic, St Olav University Hospital, Trondheim, Norway;; 8 Department of Research and Innovation, Division of Clinical Neuroscience, Oslo University Hospital, Oslo, Norway;; 9 Department of Neurology, Oslo University Hospital, Oslo, Norway; and; 10 HUNT Center for Molecular and Clinical Epidemiology, Department of Public Health and Nursing, Faculty of Medicine and Health Sciences, Levanger, Norway.

## Abstract

**Background and Objectives:**

In the absence of biomarkers, the true biological footprint of migraine remains incompletely understood. It could perhaps be best characterized using machine learning models of multimodal data. The aim of this study was to (1) develop diagnostic models of migraine using multimodal data and (2) identify data-driven migraine phenotypes.

**Methods:**

This was a cross-sectional machine learning analysis of demographics, self-reported clinical and headache data, and genome-wide genotype data from the Trøndelag Health Study (data collected 1995–1997 and 2006–2008). All participants who were genotyped and completed the headache questionnaire were included. First, predictive machine learning models were developed using genotype data and general clinical data (excluding headache data) to diagnose individuals with migraine vs headache-free controls. Models were optimized on a training set and evaluated on a held-out test set, scored with the area under the receiver operating characteristic curve (AUC). Second, unsupervised models were trained on the headache data and the most predictive features from the diagnostic models to identify subgroups. The subgroups were compared using genome-wide association analyses, conventional polygenic risk scores (PRSs), and machine learning–based genetic risk scores.

**Results:**

A total of 43,197 individuals were included in the diagnostic models, and 12,185 individuals were included in the data-driven phenotyping (mean [SD] age 49.1 [16.7] years; 51.7% women). The top-performing diagnostic model was a light gradient boosting machine, with a test set AUC of 0.80 (95% CI 0.78–0.81). Two main clusters were identified, one with 1,425 individuals, 94% of whom met diagnostic criteria for migraine, and another with 10,760 individuals, whereof 71% had nonmigraine headaches. The former was subclustered into 4 relatively distinct groups: one with only men, one with prominent neck pain, one with more musculoskeletal pain, anxiety and depression, and one with “classic” migraine. The groups were better discriminated by machine learning–based genetic risk scores compared with PRSs.

**Discussion:**

Migraine can accurately be diagnosed from nonheadache data, suggesting that it is biologically describable by combinations of clinical, genetic, and environmental data. Data-driven phenotyping with such data identifies migraine subgroups with distinct phenotypic and genotypic signals, possibly not captured by current diagnostic criteria—but with potential implications for management.

## Introduction

Migraine has no established causation or definitive biomarkers, and the diagnosis is clinical, based on the presenting phenotype of the headache and its associated symptoms, as delineated by consensus diagnostic criteria.^1^ It is characterized by recurrent episodes of intense, pulsating, and unilateral headaches, frequently accompanied by nausea, vomiting, and heightened sensitivity to light and sound.^1^ This clinical constellation is presumed to reflect a relatively homogeneous biological entity. Still, the underlying pathophysiology remains incompletely understood. Migraine is believed to arise from a complex interplay of genetic and environmental factors and demonstrates strong heritability.^2,3^ Yet, genome-wide association studies (GWASs) to date explain only a small proportion of the heritability.^4^

This discrepancy may be attributed to nonadditive genetic interactions^5^ and gene-environment interactions.^6^ Although polygenic risk scores (PRS) can be used to estimate the additive risk of complex traits, they do not currently account for higher order interactions,^7^ rendering genetics an incompletely derived biomarker. Our recent work suggests that complex modeling of genotype data can capture nonadditive interactions, outperforming polygenic risk scoring.^8^

Furthermore, the etiologic and diagnostic relevance of comorbid disorders and external factors remains incompletely characterized. In addition, here, our data indicate that combining nonheadache clinical data and genetic data better predicts the new onset of migraine, compared with either datatype alone.^9^

Considering these heterogeneous etiologic factors, as well as the heterogeneous clinical presentations and widely variable treatment responses observed across pharmacologic therapies,^10^ it has been debated whether migraine represents a unitary biological entity, or rather a spectrum of related syndromes.^11,12^

Over the past few years, there has been a rapid increase in the use of artificial intelligence (AI) and machine learning in headache research.^13^ Recent publications have demonstrated the potential for advanced computational models building on machine learning to enhance diagnostics, treatment selection, and prognostication.^13,14^

We hypothesize that if migraine indeed represents a biologically heterogeneous entity, a complex AI approach—capable of capturing high-dimensional representations of multimodal data—could with better precision identify individuals meeting diagnostic criteria. Furthermore, that a data-driven approach to phenotyping would reveal intrinsic clusters within the data, possibly corresponding to both clinical classification and genetic architectures.

The objectives of this study were to (1) develop diagnostic models for migraine using general nonheadache clinical data in combination with genetic data and (2) identify data-driven migraine phenotypes and investigate their phenotypic and genotypic signatures.

## Methods

### General Study Design

**Figure 1:**
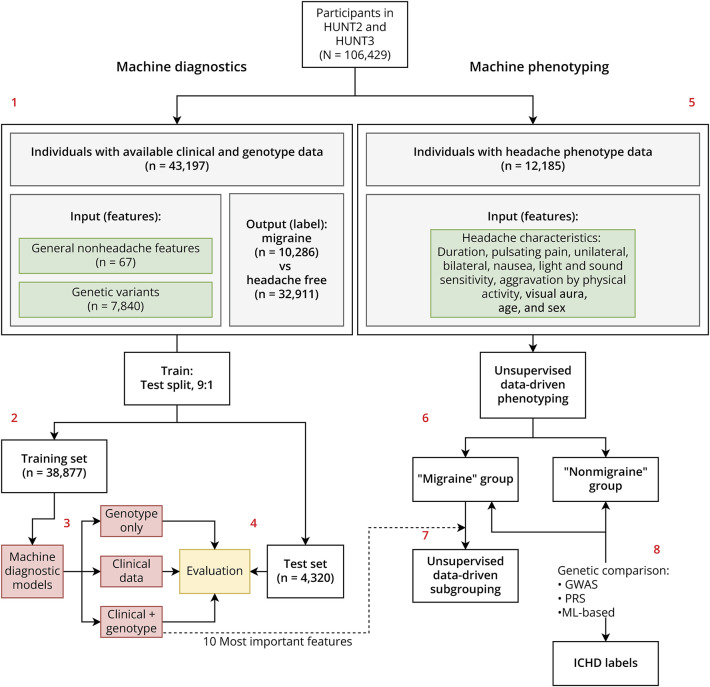
Study Overview (1) A first data set is created to develop machine diagnostic models, comprising 67 nonheadache clinical characteristics and 7,840 genetic variants. (2) The data set is split in a 9:1 ratio in training and test sets. (3) Three machine diagnostic models are created and trained on the training set: using only genotype data; using only clinical data; and using clinical and genotype data. (4) The 3 models are evaluated in the held-out test set. (5) A second data set is created for machine phenotyping, comprising individuals with headache, including headache characteristics, age, and sex. (6) Unsupervised models are used to identify one group closely resembling migraine according to diagnostic criteria and a second group with nonmigraine headaches. (7) The migraine group is broken down into subgroups based on cluster analysis of the nonheadache characteristics identified in the diagnostic models. (8) The groups identified by machine phenotyping are compared with the ICHD-labeled groups using GWASs, PRSs, and ML-based genetic comparisons. GWAS = genome-wide association study; ICHD = International Classification of Headache Disorders; ML = machine learning; PRS = polygenic risk score.

This was a cross-sectional population-based machine learning analysis of genotype and clinical data from the second and third Trøndelag Health Study (HUNT2 and HUNT3). Two main approaches were applied. First, we developed and evaluated supervised machine learning models aiming to distinguish individuals with migraine from headache-free controls, using genetic and general clinical data from HUNT2 and HUNT3 (machine diagnostic modeling). Second, we developed unsupervised representational and clustering models of clinical data and headache characteristics from HUNT2 (machine phenotyping), to identify naturally occurring subgroups in the population. The clinical and genetic characteristics of the identified subgroups were thereafter compared using GWASs and statistics. Figure 1 shows an overview of the study design.

### Data Sources and Data Materials

#### The Trøndelag Health Study

The Trøndelag Health Study is a large, population-based cohort study from Trøndelag County in Norway that has been conducted in 4 waves (HUNT1 to HUNT4).^15^ Clinical assessments, biological samples, and self-administered questionnaires were used to gather data, such as sociodemographic characteristics, lifestyle, and medical history. Clinical personnel also performed physical examinations and took blood samples. Peripheral venous blood samples were collected from participants during HUNT2 (1995–1997, n = 65,228) and HUNT3 (2006–2008, n = 50,800).^16^ Genomic DNA was extracted from whole blood–derived material (buffy coat) and stored in the HUNT Biobank under standardized conditions. For individuals with samples from both HUNT2 and HUNT3, a single genomic DNA sample per individual was used for genotyping.^16^

#### Phenotype Assignment for Diagnostic Models

A diagnosis of migraine was assessed using a modified version of the first and second International Classification of Headache Disorders (ICHD)^17,18^ based on the questionnaires in HUNT2 and HUNT3. Participants were asked whether they had experienced headache during the past 12 months, and those who answered “yes” were classified as headache sufferers, whereas those who answered “no” constituted the control group of headache-free individuals. Those answering “yes” were subsequently asked questions about their headache to assess whether they had migraine. Because the HUNT headache questionnaires did not directly correspond with the ICHD criteria, the migraine diagnosis was operationalized as a presumed diagnosis based on characteristics closely resembling the criteria. The following 3 criteria had to be fulfilled: headache lasting 4–72 hours (<4 hours was acceptable in cases where visual aura was reported); headache with at least one of the following characteristics: pulsating quality, unilateral location, or aggravation by physical activity; and at least one of nausea, photophobia, or phonophobia during headaches. Participants were also asked whether they experienced migraine, and those responding positively were labeled as having migraine. This method of phenotype assignment has been validated,^19,20^ with a sensitivity of 67%–69% and a specificity of 89%–96%. Similarly, the reliability of the self-administered musculoskeletal questionnaire has shown evidence of satisfactory performance.^21^

#### Genotyping

A total of 71,680 individuals, who participated in HUNT2 and/or HUNT3, were genotyped at the Genomics-Core Facility of the Norwegian University of Science and Technology using 3 different versions of the Illumina HumanCoreExome microarray.^16^ The HumanCoreExome microarray reflects the state-of-the-art genotyping technology available at the time of HUNT2 and HUNT3 but provides lower genomic coverage than contemporary high-density arrays or sequencing-based approaches. The complete methods for imputation and quality control of genotype data are described in detail elsewhere.^22^ In brief, after rigorous quality control, genotypes were imputed using a customized reference panel consisting of the Haplotype Reference Consortium release 1.1. Finally, variants with imputation quality *r*^2^ < 0.3 were excluded.

### Data Management and Preprocessing

Two data sets were constructed to address the first and second aim of the study, correspondingly (Figure 1 shows a schematic overview):

1. The first data set was used for distinguishing migraine from headache-free controls using supervised machine learning (machine diagnostic modeling). This data set comprised genotype data and sociodemographic and general nonheadache clinical data from HUNT2 and HUNT3.
2. The second data set was used for unsupervised clustering (machine phenotyping) and comprised self-reported clinical data and headache characteristics from HUNT2.

#### Machine Diagnostic Model Data Set

Among all available variables in HUNT2 and HUNT3, those that had a known or plausible relationship with migraine were considered for inclusion in the machine diagnostic models. This included data on demographics; socioeconomic status; work and education; history of familial diseases, as well as cardiovascular, gynecologic, neurologic, and musculoskeletal diseases; mental health; use of stimulants such as alcohol and nicotine; use of selected medication; exercise; and sleep patterns (eTable 1 provides a complete description of all included variables). Because HUNT2 generally had lower degrees of missingness and higher clinical validity, data from HUNT2 were used whenever available. In cases where there were no available data in HUNT2, the corresponding variable from HUNT3 was used. Because there were some inconsistencies in how variables were collected in HUNT2 and HUNT3, HUNT3 variables were converted to match those of HUNT2. The label (migraine or headache-free) was defined by the above-described method of phenotype assignment using data from the same questionnaire.

The following strategy was used to handle data missingness. First, we decided whether a feature had missing data completely at random, missing data at random, or missing data not at random. Features missing data not at random were generally imputed manually based on semantical logic of HUNT questionnaire's structure; for example, men did not answer questions that were aimed for women. For features missing data completely at random and at random, the multivariate imputation by chained equations (MICE) approach^23^ was used to impute data. We have previously shown that MICE works well for this data set.^9^ Details on the strategies for imputation are summarized in eTable 2.

The choice of genotype data to include in the diagnostic models was based on our previous work^8^ and is summarized here: We used the complete, genome-wide summary statistics from a 2022 GWAS meta-analysis of migraine.^4^ Because HUNT participants were part of this GWAS meta-analysis,^4^ a reverse meta-analysis procedure was conducted to derive a new beta coefficient and standard error for each variant after excluding individuals from the HUNT study. Using the recalculated beta and standard error, updated *p* values for the migraine association were obtained by calculating the cumulative density function of a normal distribution, with a mean of 0.0 and a SD of 1.0. Finally, we selected the variants reaching a significance level of *p* < 1 × 10^−5^.

#### Machine Phenotyping Data Set

To create the data set for machine phenotyping, we used the self-reported headache characteristics in the HUNT questionnaires. These included headache duration, pulsating pain, unilateral headache, bilateral headache, nausea, light and sound sensitivity, aggravation by physical activity, and visual migraine aura. In addition, age and sex were included in the machine phenotyping data set. Only patients with a complete list of the aforementioned variables were included in the data set. We chose not to impute data because these variables were also used to inform the ground truth (i.e., the phenotype assignment as described above), which potentially could lead to data leaks.

### Machine Diagnostic Modeling

The input features for the machine diagnostic modeling were the general nonheadache clinical data from HUNT2 and HUNT3 as described above and the genotype data as described above. Of note, the headache characteristics were not used as input features for the diagnostic models. The phenotype assignment as described above (migraine vs headache-free) was used as the outcome (label). The data were split in a random fashion, stratified on the label, into a training set and a test set in a 9:1 ratio. The test set was the same for all models and was kept unseen until a final model was selected for test set evaluation.

Three diagnostic models were created, differentiated by their combination of features: The first used only nonheadache clinical data as input. The second used only genotype data and has been reported previously.^8^ The third used the combined nonheadache clinical and genotype data.

A series of standard machine learning classification architectures were evaluated: logistic regression, least absolute shrinkage and selection operator, support vector machines, decision trees, k-nearest neighbors, naïve Bayes, random forest, gradient boosting methods, and ensemble methods. Models were trained on the training set, and performance was evaluated with 10-fold cross-validation. Model hyperparameters were optimized using the AutoML optimization approach. The area under the receiver operating characteristic curve (AUC) was used as a scoring metric for training and optimizing the models. The mean AUC and its SD were calculated across the 10 training folds to summarize the model's training performance and the variability between folds. In addition to AUC, we calculated accuracy, precision, recall, and macro-averaged F1 score. The top-performing model for each of the 3 data sets was finally applied on the test set to quantify out-of-sample performance, calculating AUC with bootstrapped 95% CIs. All machine learning analyses were conducted using Python 3.10 (Python Software Foundation) with open-source packages (eTable 3).

#### Model Explainability

For the top-performing clinical data–only diagnostic model, we calculated Shapley values and constructed a Shapley Additive exPlanations (SHAP) summary plot. SHAP is a framework using Shapley values to explain machine learning model predictions. SHAP assigns each feature an importance value, which enables interpretation of how much the feature contributes toward the prediction. We also conducted a SHAP interaction analysis.

### Unsupervised Machine Phenotyping

The headache characteristics from HUNT2, in addition to age and sex, were used as input features for machine phenotyping models. A series of unsupervised clustering models were used to identify subgroups with similar data patterns, including K-means, bisecting K-means, spectral, agglomerative, affinity propagation, mean-shift, and Gaussian-mixture. Cluster quality was evaluated using the Silhouette score and Davies-Bouldin index.^24,25^

We then used the Uniform Manifold Approximation and Projection (UMAP) dimensionality reduction algorithm^26^ to visualize high-dimensional data by projecting them into 2-dimensional embeddings on scatterplots. UMAP estimates the topological structure of high-dimensional data and uses this information to convert it to a low-dimensional representation that preserves both local and global relationships in the data. A grid-search strategy was used to decide hyperparameters for the UMAP analysis.

Further subclustering of the initially identified clusters was conducted using the machine phenotype data set, as well as the 10 most important features identified in the diagnostic models. A similar modeling approach to the above was used. The identified subclusters were compared statistically using multivariate analysis of variance tests.

#### Genetic Comparison of Data-Driven Phenotypes

Three strategies were used to evaluate and compare the genetic architecture of the identified data-driven clusters with those labeled as migraine according to the modified ICHD criteria.

First, we conducted separate GWASs of those labeled as migraine by the machine phenotyping vs those labeled as migraine by the modified ICHD-based phenotype assignment. SAIGE with default parameters^27^ was implemented on selected genetic risk variants, provided by the largest available migraine GWAS meta-analysis.^4^ GWAS results were used to construct Manhattan plots. To compare the results of the 2 different GWAS analyses, we (1) selected the top variants from each significant locus in each of the GWASs, using *p* value thresholds of 5e-8 and 1e-5, requiring minor allele frequency ≥0.01 and including only the top variant from each significant locus within 250-kb sections and (2) constructed QQ and cluster plots comparing the effect sizes (β) of the variants in the 2 GWAS analyses, respectively.

Second, we calculated the mean PRS for each cluster identified in the machine phenotyping. PRSs were calculated using PLINK,^28^ as previously described.^8^ PRSs were normalized to a scale of 0–1 and averaged over the clusters.

Third, we extracted the output probabilities of the previously reported machine learning–based genetic risk scoring.^8^ In this study, a light gradient boosting machine was developed using genotype data to classify individuals as having migraine vs being headache-free. The model outputs a probability on a scale of 0–1, and as mentioned above, these values were averaged over the clusters identified in the machine phenotyping.

The PRSs and machine learning–based genetic risk scores were compared across clusters using a 2-sided independent *t* test, calculating the mean difference (MD) with 95% confidence intervals. The genetic risk scores of the subclusters were compared statistically with analysis of variance tests. The significance threshold was set to 0.05. Normality assumption was based on visual inspection of histograms and QQ plots.

### Standard Protocol Approvals, Registrations, and Participant Consents

Participation in this study was based on informed, written consent, and the study was approved by the Regional Committee for Medical and Health Research (#2015/576/REK Midt and #2014/144/REK Midt), in accordance with the Declaration of Helsinki.

### Data Availability

The data sets generated and analyzed during this study are not publicly available because they contain sensitive personal information.

## Results

### Sample Characteristics

#### Demographics and Phenotype Assignment

A total of 43,197 individuals were included in the machine diagnostic modeling. Among them, 8,974 individuals (21%) were classified as having migraine and 34,223 (79%) were classified as headache-free controls (eFigure 1). Among those with migraine, 6,341 (70%) were women, and among the headache-free controls, 15,972 (46%) were women. The mean ages of the migraine cases and the headache-free controls were 42.0 (SD = 13.6) and 50.9 (SD = 16.9) years, respectively. The full population characteristics are provided in eTable 4.

A total of 12,185 individuals from HUNT2 with available phenotype data were included in the machine phenotyping (eFigure 2). A total of 4,437 individuals (37%) were classified as having migraine, and 7,748 (63%) were classified as having nonmigraine headache according to the modified ICHD criteria. Among those with migraine, 3,160 (71%) were women, and among those with nonmigraine headache, 4,712 (60%) were women. The mean ages of the migraine and the nonmigraine headache cases were 40.43 (SD = 12.06) and 41.15 (SD = 13.21) years, respectively.

### Machine Diagnostics

The top-performing model was the light gradient boosting machine, achieving a cross-validated AUC and accuracy of 0.80 (SD = 0.011). In the held-out test set, the AUC was 0.79 (95% CI 0.78–0.81; eFigure 3). The precision and recall of the held-out test set were 0.66 and 0.72, respectively, and the macro-averaged F1 score was 0.67 (95% CI 0.65–0.68).

The results of the machine diagnostics based on genotype data alone have been reported in detail elsewhere.^8^ In summary, 4 data sets with an increasing number of genetic variants were evaluated. A light gradient boosting model achieved a held-out test set AUC of 0.63 for data sets with 108; 7,771; and 7,840 genetic variants and 0.62 for a data set with 140,467 variants.

Combining the genotype data and the nonheadache clinical data resulted in a held-out test set AUC of 0.80 (95% CI 0.78–0.81). Complete metrics of all the diagnostic models are given in Table 1. Model hyperparameters are given in eTable 5.

**Table 1:** Machine Diagnostic Model Performance

	Only clinical data		Only genotype data		Combined data	
	Train, mean (SD)	Test (95% CI)	Train, mean (SD)	Test (95% CI)	Train, mean (SD)	Test (95% CI)
AUC	0.80 (0.01)	0.79 (0.78–0.81)	0.65 (0.01)	0.63 (0.62–0.66)	0.81 (0.01)	0.80 (0.78–0.81)
Accuracy	0.73 (0.01)	0.73 (0.71–0.74)	0.61 (0.01)	0.62 (0.59–0.63)	0.81 (0.01)	0.81 (0.80–0.82)
F1 score	0.67 (0.01)	0.67 (0.65–0.68)	0.58 (0.01)	0.57 (0.55–0.58)	0.70 (0.01)	0.69 (0.67–0.70)
Recall	0.73 (0.01)	0.72 (0.70–0.75)	0.60 (0.01)	0.60 (0.57–0.62)	0.68 (0.01)	0.67 (0.64–0.69)
Precision	0.66 (0.01)	0.66 (0.64–0.68)	0.60 (0.01)	0.60 (0.57–0.62)	0.71 (0.01)	0.71 (0.68–0.73)

Abbreviation: AUC = area under the receiver operating characteristic curve.

#### Model Explainability

**Figure 2:**
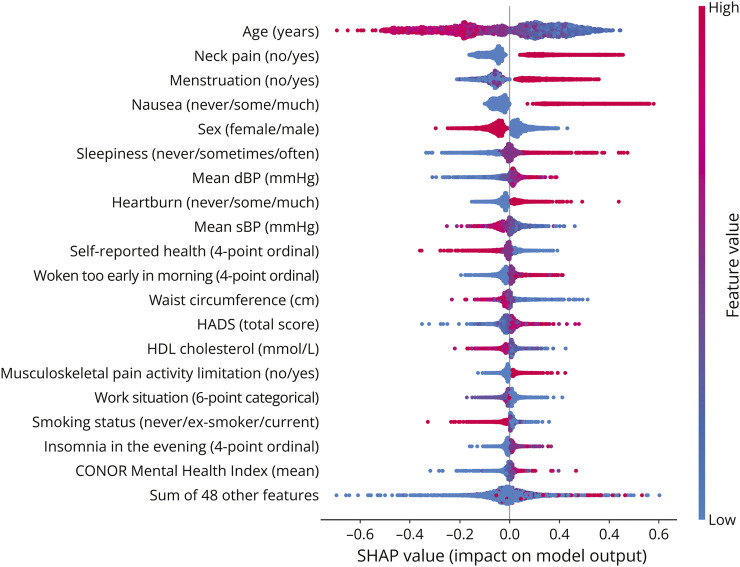
SHAP Summary Plot of the Best Predictive Model The features are ordered from the most important (top) to least important (bottom). Age is the most important predictor, followed by neck pain, menstruation, and nausea. For each feature, the impact of that feature toward having or not having migraine is plotted for each participant as a single dot. Dots placed to the right of the vertical axis predict having migraine, whereas dots placed to the left of the vertical axis predict not having migraine. Redder color of the dots indicates a higher value (i.e., high age), while bluer color of the dots indicates a lower value (i.e., low age). As an example, high age (bright red dots to the left of the vertical axis) seems to be predictive of not having migraine, while the presence of neck pain, menstruation, or nausea (red dots to the right of the vertical axis) predicts having migraine. CONOR = Cohort of Norway; dBP = blood pressure; HADS = Hospital Anxiety and Depression Scale; HDL = high-density lipoprotein; sBP = systolic blood pressure.

Based on the SHAP analysis, we found age, sex-related features (female sex and menstruation), neck pain, nausea (captured from the questionnaire on gastrointestinal symptoms), sleepiness, low systolic or high diastolic blood pressure, and good self-reported health and mental health to be the topmost important features for predicting migraine (Figure 2). The SHAP interaction analysis identified significant interactions between age, sex, menstruation, birth control pills, and self-reported health (eFigure 4).

### Machine Phenotyping

**Figure 3:**
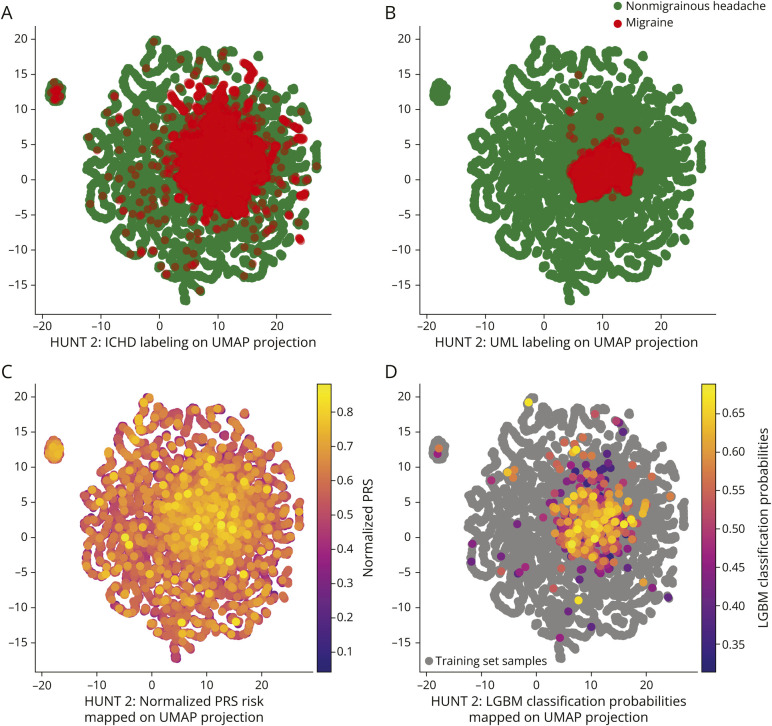
Clustering With UMAP The headache characteristics describing each participant is reduced to 2 dimensions using the UMAP algorithm. These 2 dimensions can then be visualized in scatterplots, where the x-axis represents the first latent dimension and the y-axis represents the second latent dimension. The same scatterplot is displayed in each panel, but colored using different strategies. (A) Cases are colored as migraine (red) and controls are colored as nonmigraine (green) according to modified ICHD criteria. (B) Cases are colored as migraine (red) and controls are colored as nonmigraine (green) according to the data-driven phenotyping approach. (C) All participants are colored according to their PRSs. Brighter color (yellow) indicates a higher PRS. There seems to be an increased concentration of high PRSs in the same area as those labeled as migraine according to modified ICHD criteria. (D) All participants of the test set are colored according to their complex machine learning–based nonadditive genetic risk scores. This seems to produce a tighter concentration of high-risk individuals around those labeled as migraine according to the data-driven phenotyping. ICHD = International Classification of Headache Disorders; LGBM = light gradient boosting machine; PRS = polygenic risk score; UMAP = Uniform Manifold Approximation and Projection.

For machine phenotyping, spectral clustering with 2 clusters achieved the best results, identifying one cluster with 1,425 individuals whereof 94% had migraine and 6% had nonmigraine headache according to modified ICHD criteria (cluster 2) and a second cluster with 10,760 individuals where 71% had nonmigraine headache and 29% had migraine according to modified ICHD criteria (cluster 1; Figure 3; eTable 6). The phenotypic characteristics of these clusters are summarized in Table 2.

**Table 2:** Headache Phenotype Based on Cluster

Feature	Cluster 1, %	Cluster 2, %	Cluster 2.1, %	Cluster 2.2, %	Cluster 2.3, %	Cluster 2.4, %
Duration						
<4 h	59	16	23	18	15	15
4 hours–3 days	38	77	72	80	78	85
>3 d	3	7	5	2	7	0
Pulsating quality						
Seldom or never	41	13	14	15	11	12
Now and again	45	31	30	31	32	18
Often	14	56	56	54	55	70
Unilateral						
Seldom or never	77	30	34	28	29	40
Now and again	14	24	23	24	23	22
Often	9	46	43	48	48	38
Bilateral						
Seldom or never	71	41	46	35	41	42
Now and again	22	29	26	27	29	23
Often	7	30	28	38	30	34
Aggravation by physical activity						
Seldom or never	55	9	11	3	8	8
Now and again	32	22	19	23	23	18
Often	13	69	70	74	69	74
Nausea						
Seldom or never	69	12	16	7	10	10
Now and again	27	40	44	41	40	37
Often	4	48	40	52	50	53
Light sensitivity						
Seldom or never	67	3	3	4	3	3
Now and again	26	25	21	18	26	26
Often	7	72	76	78	71	71
Visual aura						
Seldom or never	84	32	31	33	33	38
Now and again	13	32	28	27	32	27
Often	3	36	41	40	35	35
Migraine as per modified ICHD criteria	29	96	92	95	94	97
Nonmigraine headache as per modified ICHD criteria	71	4	8	5	6	3

Abbreviation: ICHD = International Classification of Headache Disorders.

**Figure 4:**
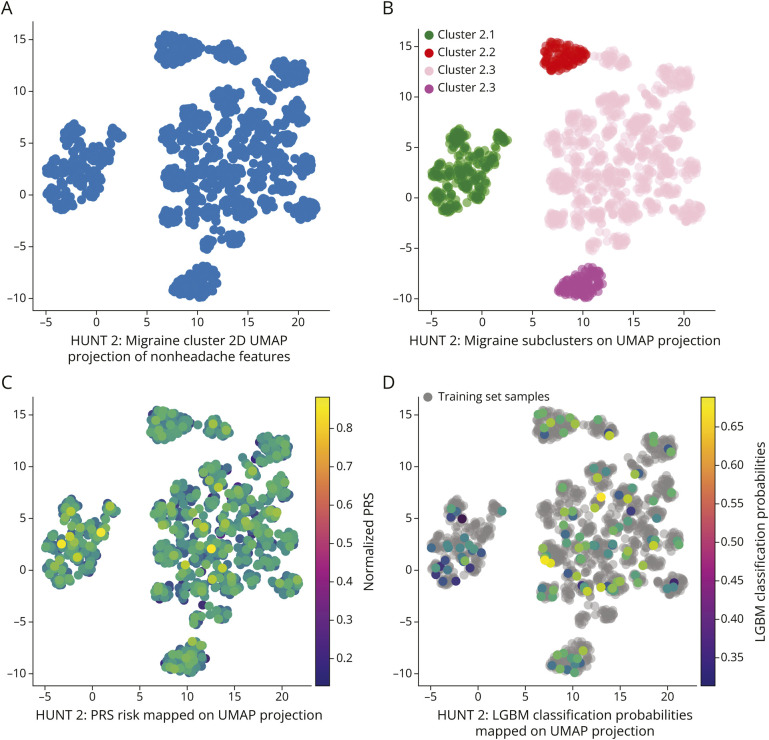
Subclustering With UMAP In this plot, participants identified as having migraine according to the data-driven phenotyping are further subclustered based on the 10 most predictive nonheadache characteristics. UMAP is used to reduce these 10 features to 2 dimensions. These 2 dimensions are then visualized in scatterplots, where the x-axis represents the first latent dimension and the y-axis represents the second latent dimension. The same scatterplot is displayed in each panel but colored using different strategies. (A) Four distinct subclusters can be identified. (B) The most distinctive subclusters are colored. (C) The participants are colored according to their PRS. Brighter color (yellow) indicates a higher PRS. (D) The participants are colored according to their complex machine learning–based nonadditive genetic risk scores. Brighter color (yellow) indicates a higher score. LGBM = light gradient boosting machine; PRS = polygenic risk score; UMAP = Uniform Manifold Approximation and Projection.

Further attempts at subclustering cluster 2 using only the headache characteristics did not identify any clear subclusters. The 2-dimensional UMAP plotting produced no visually evident clusters, and there was no statistically significant difference in the headache features between these clusters (*F*_(10, 1,414)_ = 1.75, *p* = 0.065). However, further subclustering using the 10 most important features from the machine diagnostic model revealed 4 distinct subclusters (clusters 2.1 through 2.4, Table 3). These clusters were statistically significantly different (*F*_(36, 4,236)_ = 105.63, *p* < 0.001). Cluster 2.1 comprised only men and the other 3 only women. Among the women clusters, cluster 2.2 was characterized by neck pain and the absence of general gastrointestinal symptoms. Cluster 2.3 was characterized by general musculoskeletal pain, lower self-reported health, and higher Hospital Anxiety and Depression Scale (HADS) scores. The last cluster, 2.4, was characterized by lower HADS scores and pulsating pain. Figure 4 shows the UMAP morphology of the clusters, and Tables 2 and 3 provide the phenotypic characteristics.

**Table 3:** Characteristics of the Most Predictive Features

Feature	Cluster 1	Cluster 2	Cluster 2.1	Cluster 2.2	Cluster 2.3	Cluster 2.4
Age, y, mean	40.94	40.54	41.09	39.69	40.78	37.11
Sex (female), %	62	82	0	100	100	100
Menstruation (yes), %	47	63	0	100	73	100
Nausea, %						
Not at all	79	64	71	100	57	57
Slightly	18	30	23	0	38	34
Very much	3	6	6	0	5	9
Neck pain, mean	0.36	0.50	0.46	1.00	0.51	0.0
Contraception (yes), %	10	11	0	0	15	0
HADS score, mean	4.77	5.62	5.81	5.23	5.71	4.43
Musculoskeletal pain (yes), %	51	62	60	0	66	0
Systolic BP, mean (mm Hg)	130.7	128.6	133.7	126.5	128.1	122.7
Self-reported health, mean	1.91	1.73	1.68	1.99	1.69	2.0
Mental health, mean	1.56	1.65	1.69	1.55	1.66	1.51
Heartburn, %						
Not at all	65	62	54	100	57	100
Slightly	28	29	31	0	34	0
Very much	7	9	15	0	9	0
Migraine as per modified ICHD criteria, %	29	94				
Polygenic risk score, mean (SD)	0.47 (0.11)	0.48 (0.11)	0.49 (0.12)	0.48 (0.10)	0.48 (0.11)	0.48 (0.13)
Complex nonadditive genetic risk score, mean (SD)	0.51 (0.08)	0.53 (0.08)	0.46 (0.06)	0.56 (0.06)	0.54 (0.07)	0.53 (0.06)

Abbreviations: BP = blood pressure; ICHD = International Classification of Headache Disorders.

For each cluster, the percentage meeting modified ICHD criteria, the mean polygenic risk score, and the mean complex non-additive genetic risk scores are calculated.

#### Genetic Comparison

Genotype data from a total of 44,021 individuals were used to conduct GWASs, with 39,584 headache-free controls and 4,437 with migraine included in the GWAS based on modified ICHD criteria labeling and 42,596 headache-free controls and 1,425 with migraine included in the GWAS based on unsupervised labeling. The Manhattan plots are shown in eFigure 5. There was a high degree of overlap in the identified risk loci (eFigure 6).

The mean PRS was 0.471 (SD = 0.109) in cluster 1 and 0.478 (SD = 0.113) in cluster 2, with a MD of 0.007 (95% CI 0.00–0.014; *p* = 0.034; n = 10,947). The PRSs were not statistically different between the subclusters (*F*_(3, 1,283)_ = 0.77, *p* = 0.51).

The average machine learning–based genetic risk score for cluster 2 was 0.526 (SD = 0.077) compared with 0.512 (SD = 0.083) for cluster 1. The MD in scores was 0.019 (95% CI 0.014–0.025; *p* < 0.001; n = 4,599). The machine learning–based genetic risk scores were significantly different between the subclusters (*F*_(1, 1,285)_ = 247.78, *p* < 0.001).

## Discussion

Because migraine is still a criterion-defined disorder without known biomarkers, scientists and clinicians have debated whether it represents a unitary biological entity, or rather a collection of related syndromes,^11,12^ that is, a *migraine spectrum disorder*. In this study, we used a range of AI-based approaches to evaluate the biological coherence of migraine, asking whether it is a “true” disorder in a definable space of clinical, biological, and genetic features.

It is plausible that migraine's biological signature is distributed in a high-dimensional space that could best be captured by machine learning. Our findings support this notion. Even in the absence of headache-specific features, our diagnostic models achieved high diagnostic performance (AUC 0.79), suggesting that migraine is associated with a wider constellation of clinical and biological markers. This may even support a reconceptualization of migraine as a spectrum disorder that can be identified and stratified using broad clinical and biological data—not solely through headache-specific symptoms.

Yet, the minimal improvement achieved by adding genetic data (AUC 0.80) raises an important question: if migraine can be described by a high-dimensional set of biomarkers, both genetic and clinical, why does the inclusion of genetic information provide only marginal improvement in accuracy? One likely explanation is that rich clinical data already capture much of the downstream manifestations of genetic risk, effectively absorbing its predictivity. While genetics may play a central etiologic role, its utility for diagnosis may be limited when clinical data are available. In addition, the validity of the phenotype assignment (estimated AUC of 0.86–0.89) places a ceiling on achievable model performance, meaning that an AUC of 0.80 is close to the methodological limit in the settings of this study.^19,20^

Our data-driven phenotyping further supports the notion of migraine as a spectrum defined by a high-dimensional space of clinical and biological markers. The data-derived group appearing most similar to migraine as defined today (cluster 2) identifies the individuals with the strongest “migraine signals”—those with both the highest genetic load and the most typical migraine symptomatology. In other words, cluster 2 had a very high positive predictive value measured against the ICHD criteria, with very few false positives. This can be illustrated in Figure 3, where individuals with higher genetic load and migraine symptomatology are toward the center of the cluster. Moving further to the periphery of the cluster shows a lower genetic load and a drift toward a nonmigraine phenotype.^29^ Indeed, this is where we find many of those “wrongly” mapped to the nonmigraine cluster who have migraine according to ICHD criteria. These are individuals that could be said to not be at the center of the spectrum, that is, with less strong “migraine signals,” but still meet diagnostic criteria. Moreover, applying the machine learning–based genetic scoring, the distinction between the presumed migraine cluster (cluster 2) and nonmigraine cluster (cluster 1) becomes even more apparent, underpinning the likely nonadditive genetic architecture we have previously suggested.^8^

Further clustering of the data-derived migraine group (cluster 2) revealed interesting findings. No meaningful subgroups emerged when clustering was based solely on headache characteristics—perhaps suggesting that traditional clinical criteria alone are insufficient for identifying biologically relevant subtypes. By contrast, clustering based on the top predictive nonheadache features identified interpretable and distinct subgroups. Cluster 2.2 was defined by neck pain and absent general gastrointestinal symptoms, possibly pointing toward a cervicogenic component in a subset of patients.^30^ Cluster 2.3, characterized by musculoskeletal pain, neck pain, high anxiety and depression scores, and poor self-reported health, may represent a phenotype with central sensitization and comorbid psychiatric disorders.^31^ Indeed, a very strong relationship between migraine and the combination of high anxiety and depression scores, musculoskeletal pain, and gastrointestinal symptoms has previously been demonstrated.^32^ This is further supported by the heightened sensitization and worse psychological burden that has been seen in migraine with prominent neck and musculoskeletal pain.^33^ Cluster 2.4, distinguished by lower anxiety and depression scores and a predominance of pulsating pain, may represent the phenotype most resembling “classic” migraine according to the ICHD criteria,^1^ with minimal neuropsychiatric comorbidity. These patterns suggest that nonheadache clinical features may carry important diagnostic, mechanistic, and therapeutic information.

Other works on data-driven phenotyping of migraine are comparable to our findings. In the Chronic Migraine Epidemiology and Outcomes study, a web-based survey was used to characterize the comorbidities of individuals with migraine.^34^ Latent class analysis identified 8 natural subgroups based on comorbidities. The group with the most comorbidities also had higher degrees of disability, allodynia, and medication overuse, perhaps similar to cluster 2.3 in our analysis. Another study used a clinical data set and data reduction methods to identify naturally occurring clusters.^35^ In addition, here, a group with high levels of psychiatric comorbidity and migraine-related disability was identified. Finally, a study of Australian twins used headache data to derive subgroups based on ICHD-like symptoms and a latent class analysis.^36^ Those identified as migraine by the latent class analysis had a higher genetic contribution, similar to our findings.

Overall, our findings support the potential of machine learning to identify a high-dimensional biological migraine footprint and to uncover latent subtypes. Under this assumption, clinical diagnosis based on current criteria may not sufficiently reflect underlying biological differences among groups—differences that could have implications for disease course and treatment response. If this is true, one could raise the argument that deeper phenotyping in the clinical setting, beyond the current diagnostic criteria, could in turn improve our management of migraine. To clarify this, more research is needed. Future works should further evaluate and characterize the data-driven migraine subtypes, especially regarding disease course and therapeutic responses. Moreover, future works should consider collecting data and digital biomarkers and time-series data using wearable devices and smartphone apps to further facilitate accurate phenotyping.^37-39^

This study has several strengths. First is the rigorous machine learning approach with appropriate test set validation. Second, the diagnostic models do not suffer from the data leaks that occur in typical headache diagnostic models that incorporate headache characteristics as input features.^13^ Several weaknesses must be acknowledged. First is the lack of validation of several self-reported conditions in the HUNT questionnaires. Second, we cannot rule out misclassification of headache diagnoses. Especially, individuals with low-frequency episodic migraine might have answered “no” to the question about headache within the past 12 months and thus be falsely classified as headache-free; this is also evident from the sensitivity of 67%–69% in validation of the questionnaires. In addition, secondary headache disorders may have been misclassified. Any such limitations in validity of headache diagnoses in HUNT decrease the confidence in our fining. Third, the clinical data set is of some age (collected in 1995–2008). Many of the variables collected and used in the model, such as sleep patterns, mental health measures, hormonal factors, cardiovascular risk factors, and lifestyle behaviors have likely changed in recent decades and may not fully reflect the 2025 population landscape. Fourth, the HumanCoreExome microarray technology used is also of some age and less comprehensive than modern platforms and may make the genotypic data less reliable. Fifth, the heterogeneities identified here are correlative and do not necessarily imply causal differences. Finally, it is important to emphasize that this is a single-cohort design, which limits generalizability to other populations. The models need to be validated in an external sample before any firm conclusions may be drawn.

Migraine can accurately be diagnosed by a high-dimensional space of interactive biomarkers, which are genetic, clinical, and environmental. Within this space, clinical presentations may exist along a spectrum, potentially best informed by headache characteristics in combination with other types of data. Such high-dimensional characterization of migraine and its potential subtypes could have implications for mechanistic understanding, disease courses, and treatment responses.

## Appendix Coinvestigators

**Table:**

Coinvestigators are listed at Neurology.org/N.

## Glossary

AI: artificial intelligence
AUC: area under the receiver operating characteristic curve
GWAS: genome-wide association study
HADS: Hospital Anxiety and Depression Scale
ICHD: International Classification of Headache Disorders
MD: mean difference
MICE: multivariate imputation by chained equations
PRS: polygenic risk score
SHAP: Shapley Additive exPlanations
UMAP: Uniform Manifold Approximation and Projection
